# Amorphous titanium-oxide supercapacitors

**DOI:** 10.1038/srep35870

**Published:** 2016-10-21

**Authors:** Mikio Fukuhara, Tomoyuki Kuroda, Fumihiko Hasegawa

**Affiliations:** 1 New Industry Creation Hatchery Center, Tohoku University, Sendai, 980-8579 Japan

## Abstract

The electric capacitance of an amorphous TiO_2-x_ surface increases proportionally to the negative sixth power of the convex diameter *d.* This occurs because of the van der Waals attraction on the amorphous surface of up to 7 mF/cm^2^, accompanied by extreme enhanced electron trapping resulting from both the quantum-size effect and an offset effect from positive charges at oxygen-vacancy sites. Here we show that a supercapacitor, constructed with a distributed constant-equipment circuit of large resistance and small capacitance on the amorphous TiO_2-x_ surface, illuminated a red LED for 37 ms after it was charged with 1 mA at 10 V. The fabricated device showed no dielectric breakdown up to 1,100 V. Based on this approach, further advances in the development of amorphous titanium-dioxide supercapacitors might be attained by integrating oxide ribbons with a micro-electro mechanical system.

In recent years, much attention has been devoted to examining the quantum size effects (QSEs) that arise from the confinement of electrons in surfaces of nanometre-sized unevenness comparable to the de Broglie wavelength of electrons^1^,^2^,^3^,^4^,^5^. The QSE, which is characterized by the alteration of electronic structures, appears in various physical properties, including conductivity, surface energy, Hall effects, magnetoresistance, and chemical reactivity. We have reported that amorphous perfluoroalkenyl vinyl ether polymer (APP) surfaces with nanometre-sized convexity can store a remarkably powerful electric charge^6^.

Here, we present the convex-diameter-dependent capacitance and charging properties of a Ti-Ni-Si amorphous-alloy capacitor sandwiched by TiO_2-x_ layers with nanometre-sized surface unevenness. Amorphous TiO_2-x_ anodically oxidised using a titanium-based amorphous alloy is characterized by a higher work function (5.5 eV) than that (4.0 eV) of crystalline anatase^7^,^8^, a surface with nanometre-sized unevenness that cannot be formed from crystalline titanium alloys^9^, and electric resistance greater than 500 GΩ. In addition, the formation of uneven surfaces with round convexity is easier for titanium oxides than for aluminium ones. To our knowledge, however, no study has conducted a quantum-size analysis for the electrical capacitance and storage of an amorphous titanium oxide (ATO) surface. The de-alloyed and anodically oxidised Ti-Ni-Si alloy showed AC electric storage at temperatures ranging from 193 K to 453 K with a voltage variation from 10 V to 150 V and DC capacitances of ~4.8 F (~52.8 kF/cm^3^)^7^,^8^. We assumed a surface structure consisting of a distributed constant-equivalent circuit of resistance and capacitance; this structure is analogous to active carbons in electric double-layer capacitors (EDLCs). We termed this device a “dry” electric distributed constant capacitor (EDCC).

Since the surface analysis of the scanning Kelvin probe force microscope (SKPM) for ATO showed the adsorption of electric charge with higher work functions of 5.5 eV on its surface^7^,^8^, we prepared specimens with uneven surfaces having diameters of 4–40 nm, under various anodic oxidisation conditions in H_2_SO_4_ and (NH_4_)SO_4_-NH_4_F solutions^9^ (see Methods in the Supplementary Information for details). We first investigated the convex-diameter dependence of capacitance. Because the series capacitance shows false data when the resistance is less than 100 MΩ (see Fig. S1 in Supplementary Information), we measured the parallel capacitance of specimens with resistance greater than 1 kΩ to evaluate the QSE in capacitance. The result is shown in Fig. 1a, along with the negative-six-power fitting line for capacitance versus convex diameter. The capacitance increased remarkably with decreasing *d,* as shown in the inset of Fig. 1a, and the relationship followed an electrocapillarity formula^10^: *C* (F/cm^2^) = 2 × 10^6^*d*^−5.78^ (r^2^ = 0.963), using *C* = *εηA_0_*/*d*^*n*^, provided that the diameter is the same as the distance between two electrodes in a distributed constant-equipment circuit organizing the titanium-oxide layer. In this expression, *ε* and *A_0_* and are the permeability of free space (8.854 × 10^−12^ F/m) and original surface area of the specimen, respectively. Here, *A_0_* has the width, length, and area of 10^−3^ m, 10^−2^ m and 10^−5^ m^2^, respectively. The roughness factor *η* (128.7) was calculated using *η* = A/A_0_ = A_0__*_t_*ρ*_A_BET_/*A_0_*, where *t, ρ*, and *A*_*BET*_ are the thickness (200 nm), density (3.878 g/cm^3^)^7^, and BET specific surface area (165.9 m^2^/g) of the titanium- oxide layer corresponding to *d* = 35.4 nm, respectively.

The negative-sixth-power diameter dependency is known as the van der Waals force, which is based on induced dipole-dipole interactions^11^. This result resembles the hypothesis that the weak adhesive force exerted by the feet of many types of geckos is the result of van der Waals intermolecular forces^12^,^13^. Figure 1b,c present scanning electron microscope (SEM) images of the surface structure corresponding to values of *d* = 35.4 nm and 4.7 nm, respectively. Figure 1d shows a three-dimensional AFM image of the surface structure corresponding to *d* = 35.4 nm. The surface structure resembles that of APP (see Fig. S2 in Supplementary Information). Thus, the use of the ATO surface with unevenness less than 5 nm in diameter shows promise for the production of large EDCCs.

To analyse non-destructively the electrostatic contribution of the specimen, we measured the AC impedance from 0.1 Hz to 1 MHz at room temperature by using a device (inset of Fig. 2c) fabricated by a micro-electro mechanical system (MEMS) method (see Fig. S3 in Supplementary Information). A complex-plane plot of the impedance data obtained from the specimen for *d* = 35.4 nm is shown in Fig. 2a. The data fit a near-vertical line in the Nyquist plot, as produced by a series-*RC* circuit, as well as a graphene EDLC^14^. There were rapid increases in the imaginary impedance, as compared with the real impedance, in the lower-frequency region (Fig. 2b). Moreover, the capacitive behaviour (near the −90° phase angle) throughout the frequency region (Fig. 2c) is clear evidence for series-*RC* circuit. Thus, the ATO offers a nearly ideal electric distributed-constant structure for enhancing electrical power storage. Although the electrical storage is not always the same as the capacitance, the value of the series capacitance was 4.17 μF (2.085 F/cm^3^, 537.6 μF/kg) at 0.1 Hz (Fig. 2d).

Since decreases in the convex diameter by solvent F ions in anodic oxidisation accompanies decreases in resistance, that is, leakage of electrical charge between electrodes by the formation of penetrating tunnels in the oxide layer^9^, we oxidised the specimens for 36 ks in air to prevent the leakage of charge due to stopping of the tunnels by using specimens anodically oxidised at 10 V and 278 K for 1.8 ks in (NH_4_)_2_SO_4_-0.05 wt%NH_4_F solution. Figure 3a shows both the resistance variation for the annealing temperature and the discharging time with a constant current of 1 pA after charging with a DC current of 1 mA for 300 s. The resistance increased to 700 GΩ as the temperature increased to 448 K and then decreased to 330 GΩ at 548 K. The increase suggests the filling up of the nanometre-sized tunnels by the diffusion of oxygen atoms, while the decrease might be due to the formation of cracks. The discharging-time curve resembles the resistance curve, except for the maximum discharging time at 473 K. Thus, we selected an annealing temperature of 473 K for ATO specimens with diameters in the range of 5–10 nm and electric resistance greater than 500 GΩ. Figure 3b shows typical results for DC charging and discharging behaviours of specimens with a surface area of 76 cm^2^ under constant currents of 1 pA, 1 nA, 1 μA, and 1 mA, after charging with a DC current of 1 mA for 300 s. These results indicate the electrical power storage performance of the ATO. To provide visible evidence of the ATO electric storage, we attempted to illuminate a red LED. The device, composed of parallel combinations of *RC* circuits (see Fig. S4 in Supplementary Information), discharged up to 15 s at constant current of 1 nA and powered the LED for 37 ms (Fig. 3c), after the device was charged with 1 *μ*A for 300 s at 10 V. Figure 3d presents an illumination photograph of the LED. Further gains might be attained by integrating oxide ribbons with the MEMS.

Finally, we consider the origin of the electric storage in ATO. Figure 4a shows a microscopic schematic representation proposing a possible mechanism for large electrical charges, along with the electric-field strength and potential change from positive to negative electrodes. The electrically negative convex portion of ATO and the electrically positive concave one form many electrode double layers perpendicular to the electrode. This is a capacitor with an electric distributed-constant circuit (Fig. 4c) as well as the APP^6^. Here, we note that the atomic structure of nanometre-sized ATO has a highly distorted shell and a small, strained anatase-like core with a coordination number of 5.3 for an average Ti-O^15^. Relative to crystalline TiO_2_ with a Ti coordination number of 6, the reduction of the coordination number, which is primarily due to the truncation of the Ti-O octahedral (Fig. 4e), corresponds to an oxygen vacancy of 11.7 at.% (Fig. 4b). The vacancies, therefore, serve as the electrically positive offset effect for trapping electrons at convex surfaces on the amorphous structure (Fig. 4b), resulting in large electron adsorption. Analogous to the APP with superior electric storage resulting from the quantum-size effect, the convex-diameter dependencies of the calculated electrostatic potential and the induced outer-electron pressure of titanium atoms that occupy the centre of the ATO structure are presented in Fig. 4d by means of the electronic screening theory (discussed at length in Supplementary Information). The decrease in diameter increases both the negative potential and positive pressure. The calculated potential was consistent with the experimental data (5.5 eV)^7^ with a convexity 35.4 nm in diameter. The QSE in this study resembles space-charge polarization.

In conclusion, anodically oxidised surfaces present two advantages for the direct electron charging of giant capacitors. The first is the introduction of van der Waals attractive forces for electron adsorption by homogeneously distributed nanometre-sized convexities on amorphous TiO_2-x_ surfaces. The second is the offset effect of electron repulsion caused by the appearance of positive charges at a large number of oxygen vacancies in the amorphous TiO_2-x_ layer. The integrated ATO device, a supercapacitor, succeeded in illuminating an LED for 37 ms.

A supercapacitor with an amorphous TiO_2-x_ surface, as fabricated and tested, demonstrates a nearly ideal electric distributed-constant structure for enhancing electrical power storage. The results of tests indicated that the use of an ATO surface with unevenness maintained at less than 5 nm in diameter shows promise for the production of large EDCCs. These results indicate the potential for making further advances in the fabrication of amorphous titanium dioxide supercapacitors by integrating oxide ribbons with MEMS.

## Methods

The ATO specimens having uneven surfaces with convex diameters in the range of 3 nm to 40 nm were prepared under various anodic oxidization conditions in H_2_SO_4_ and (NH_4_)SO_4_-NH_4_F solutions. The integrated ATO device was fabricated using many ATO specimens annealed in air at 473 K for 36 ks. *I-V* and *R-V* characteristics were measured at DC voltages ranging from 0 V to 1,100 V at a sweep rate of 1.24 V/s by using a Precision Source Measure Unit (B2911A, Agilent). AC impedance and charging/discharging behaviours of the specimen were measured, using a potentiostat/galvanostat (SP-150, BioLogic Science) at room temperature.

## Additional Information

**How to cite this article**: Fukuhara, M. *et al*. Amorphous titanium-oxide supercapacitors. *Sci. Rep.* **6**, 35870; doi: 10.1038/srep35870 (2016).

## Supplementary Material

Supplementary Information

## Figures and Tables

**Figure 1:**
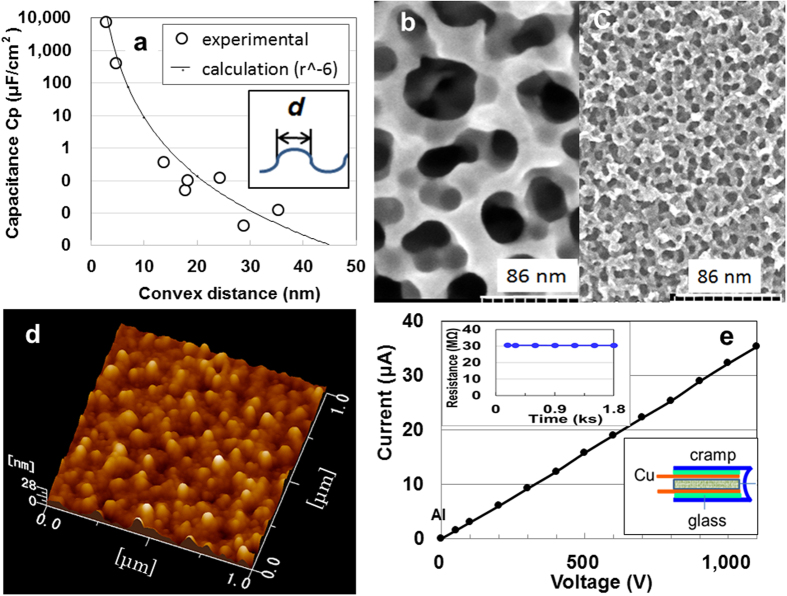
Dependence of capacitance on convex diameter *d*. (**a**) Capacitance for de-alloyed and anodic oxidized Ti-15 at.%Ni-15 at.% Si. SEM images (**b**) and (**c**) of specimens obtained by anodic oxidation in 0.4 M (NH_4_)_2_SO_4_ and 0.05 wt.% NH_4_F at 30 V for 1.8 ks, and in 0.2 M (NH_4_)_2_SO_4_ and 1.7 wt.% NH_4_F at 5 V for 1.8 ks, respectively. The three-dimensional AFM image (**d**), and *I-V* and *R-t* characteristics up to 1,100 V (**e**) and for 1.8 ks at 1,100 V (upper inset of (**e**)), respectively, of the ATO surface structure corresponding to *d* = 35.4 nm.

**Figure 2:**
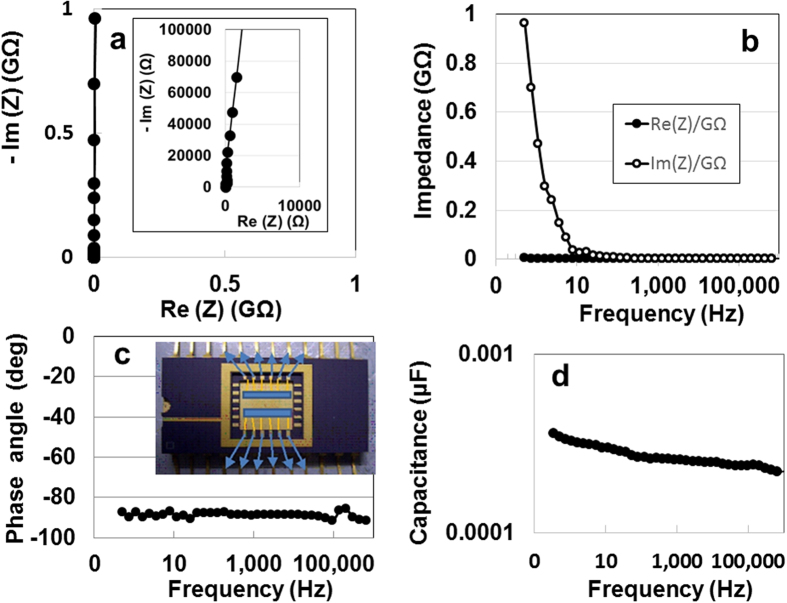
Non-destructive analysis of the electrostatic contribution of the specimen. (**a**) Nyquist plot as a function of frequency for ATO device. (**b**) Real and imaginary impedances. (**c**) Phase angle. The inset of (**c**) depicts a device fabricated by MEMS. (**d**) Series capacitance.

**Figure 3:**
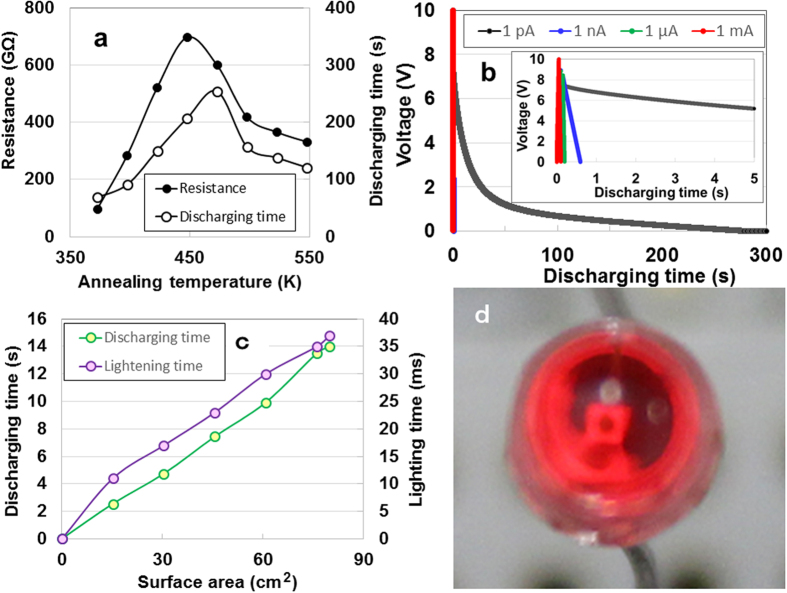
Performance measures of the ATO device fabrication. (**a**) Annealing- temperature dependencies of electrical resistance and discharge time at a constant current of 1 pA after charging at a DC current of 1 mA for 30 s for anodically oxidised specimens. (**b**) The charging/discharging behaviours under various constant currents after charging at a DC current of 1 mA for 30 s. (**c**) Discharging time at a constant current of 1 nA and lighting time for a parallel circuit as a function of its surface area. (**d**) An LED powered by the ATO device.

**Figure 4:**
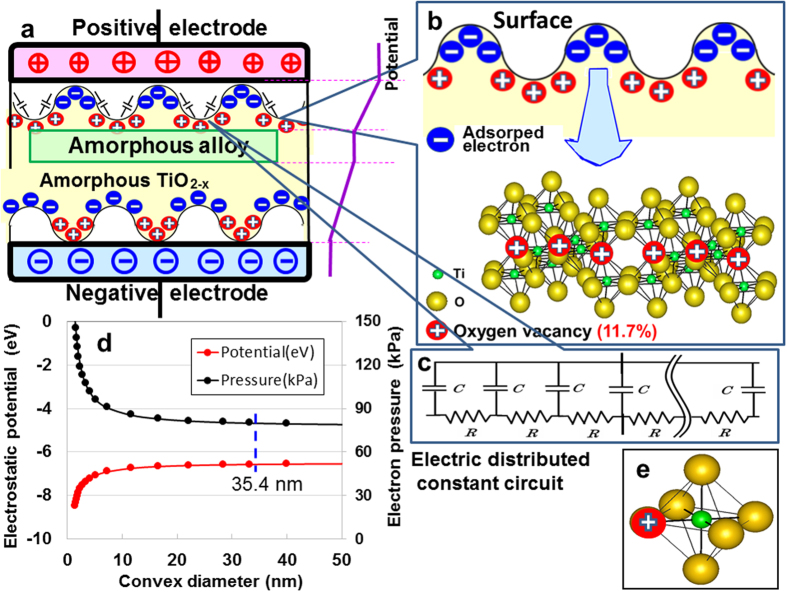
Representations of the fabricated ATO device. (**a**) The microscopic electrical energy storage. The Ti-15 at.%Ni-15 at.%Si amorphous alloy is covered with an amorphous TiO_2-x_ surface having nanometre-sized convexities. The device contains an electric double layer of electrons deposited on nanometre-sized convexities of the positively charged amorphous TiO_2-x_ surface. (**b**) Depictions of the estimated amorphous TiO_2-x_ structure with positively charged oxygen vacancies of 11.7%. (**c**) The electric distributed-constant equipment circuit organising the amorphous TiO_2-x_ surface. (**d**) Convex dependence of the electron potential and pressure for the convex portions. (**e**) A tetrahedron consisting of Ti, O, and positively charged oxygen vacancy.
