# Supplementary material for: Amorphous titanium-oxide supercapacitors
Source: Sci Rep. 2016 Oct 21;6:35870. doi: 10.1038/srep35870 (PMC5073320; doi:10.1038/srep35870)
Supplement: Supplementary Information [file srep35870-s1.pdf]

SUPPLEMENTARY INFORMATION

Amorphous titanium-oxide supercapacitors

Mikio Fukuhara [1,2] Tomoyuki Kuroda[1]& Fumihiko Hasegawa[1]

[1] New Industry Creation Hatchery Centre, Tohoku University, Sendai, Japan 980-8579

[2] Waseda University Research Organization for Nano & Life Innovation, Green Device Laboratory, Tokyo, Japan

PACS 77.22.-d, 77.84.-s, 77.90.+k, 78.55.Qr, 81.16.Pr

1. Supporting Methods

Ti-15 at.% Ni-15 at.% Si alloy was prepared by ingots (nominal compositions) were prepared by arc-melting mixtures of Ti (99.95 % purity), Si (99.999 % purity) and Ni (99.95 % purity) in Ar atmosphere purified by Ti-gettering. Ribbon specimens 50 µm thick and 1 mm wide were prepared from the ingots by rapid solidification of the melt on a single copper roller in He atmosphere at a tangential velocity of 52.3 m/s, using a single-wheel melt-quenching apparatus (NEV-A05-R10S, Nisshin Giken). For preparation of the ATO with surface roughness of 2-40 nm, the specimens were anodically oxidised at a constant potential of 5-30 V in an electrolyte of 0.4 M H_2SO_4, 0.2 M $(NH_4)SO_4$ and (0.05, 0.5 and 1.7 wt%) NH_4F solutions at 278 K, using platinum counter electrode, after de-alloying of Ni in 1 N HCl solution at room temperature for 86.4 ks. The detailed conditions for anodic oxidization are presented at Table S1.

Table S1 Eight kinds of electrolytes and anodic oxidization conditions for anodic oxidization of amorphous Ti-15 at% Ni-15at% Si alloys.

	Electrolytes	Anodic oxidized conditions	Convex distance
1	0.4M H_2SO_4	at 50V for 3.6 ks	35.4 nm
2	0.4M H_2SO_4	at 40V for 3.0 ks	28.9 nm
3	0.4M $(NH4)_2SO_4$-0.05wt% NH_4F	at 30V for 1.8 ks	24.3 nm
4	0.4M $(NH4)_2SO_4$-0.05wt% NH_4F	at 25V for 1.8 ks	18.3 nm
5	0.2M $(NH4)_2SO_4$-0.5wt% NH_4F	at 20V for 1.8 ks	17.8 nm
6	0.2M $(NH4)_2SO_4$-0.5wt% NH_4F	at 15V for 1.8 ks	13.8 nm
7	0.2M $(NH4)_2SO_4$-1.7wt% NH_4F	at 10V for 1.8 ks	4.7 nm
8	0.2M $(NH4)_2SO_4$-1.7wt% NH_4F	at 5V for 1.8 ks	2.9 nm

The specific surface area was measured by Kr adsorption, expecting that the nanometre sized porous structure significantly enhanced it, using of BET absorption (Autosorb iQ, Quantachrome) at 77 K. Sample structure was examined by X-ray diffraction (XRD) in reflection mode with monochromatic Cu Kα radiation. Fig. S1 shows X-ray pattern of the specimen after de-alloying and anodic oxidization, showing amorphous structure. Specimen surfaces were examined by field emission scanning

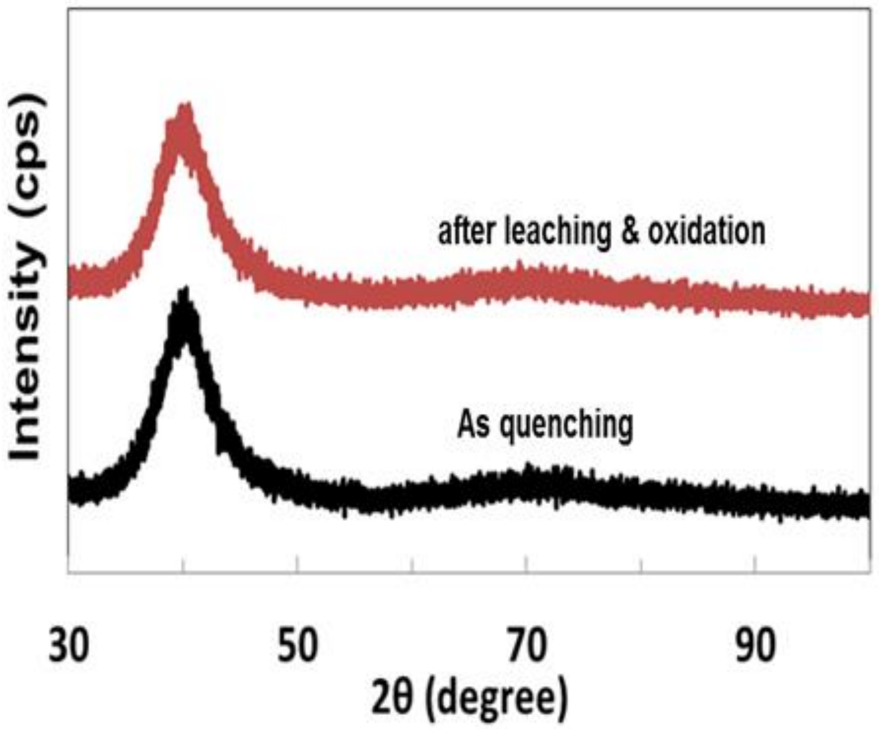

Fig. S1 X-ray diffraction patterns of the studied alloy before and after de-alloying and then anodic oxidization (after Ref. 8)

.

electron microscopy (SEM, S-5500, Hitachi) at an operating voltage of 5 kV. Topography images were observed using a noncontact atomic force microscope (NC-AFM, JSPM-5200, JEOL). However, we could not obtain the distinct three-dimensional AFM images of the ATO surface structure corresponding to d= 2.9 and 4.7 nm. Depth concentration profile of Ti, Ni, Si and O, and the three-dimensional Ti2p, Ni2p, Si2p and O1s spectra for surface of de-alloyed and then anodic oxidized specimen, using XPS microprobe (PHI-X, ULVAC-PHI) were already reported at Ref. 7.

The specimens (1 mm wide, 50 μm thick, and 10 mm long) with double–oxidized surface was sandwiched by two methods: mechanically clamp method using copper ribbons beneath two pieces of glass plates, and MEMS method by sputtering of Au/Cr onto the specimen.

The charging/discharging behaviours of each *RC* combination were analysed using the galvanostatic charge/discharge measured using a potentiostat/galvanostat (SP-150, BioLogic Science Instruments) with a DC supply of 10 V and charging currents of 1, 5, and 10 mA for approximately 3.6 ks at room temperature. All electrical measurements were carried out in an Al shield box to prevent electromagnetic interference from

influencing the results.

2. Resistance and frequency dependencies of capacitance in parallel and series circuits

To determine the capacitance accuracy of *RC* circuits, we investigated the *RC* effect after charging at a current of 1 mA and voltage of 10 V using standard resistors (10 Ω, 100 Ω, 1 kΩ, 10 kΩ, 100 kΩ, 1 MΩ, 10 MΩ, 100 MΩ and 1 GΩ0.1 Ω−1 GΩ) and ceramic capacitor of 1.0 μF in simple parallel and series *RC* circuits. The frequency dependencies of capacitance in parallel and series *RC* circuits are shown in Fig. S2**a** and **b**, respectively. In parallel *RC* (**a**), capacitance increases with decreasing frequency for lower resistance below 1kΩ. In series *RC* (**b**), on the other hands, capacitance increases remarkably for resistance below 10 MΩ, as frequency decreases. Thus we cannot evaluate capacitance in series circuit, but can do it in parallel one with resistance above 1kΩ. Since the resistances of all specimens used in this study are over 1kΩ, all capacitance data of Fig. 1**a** are truth values.

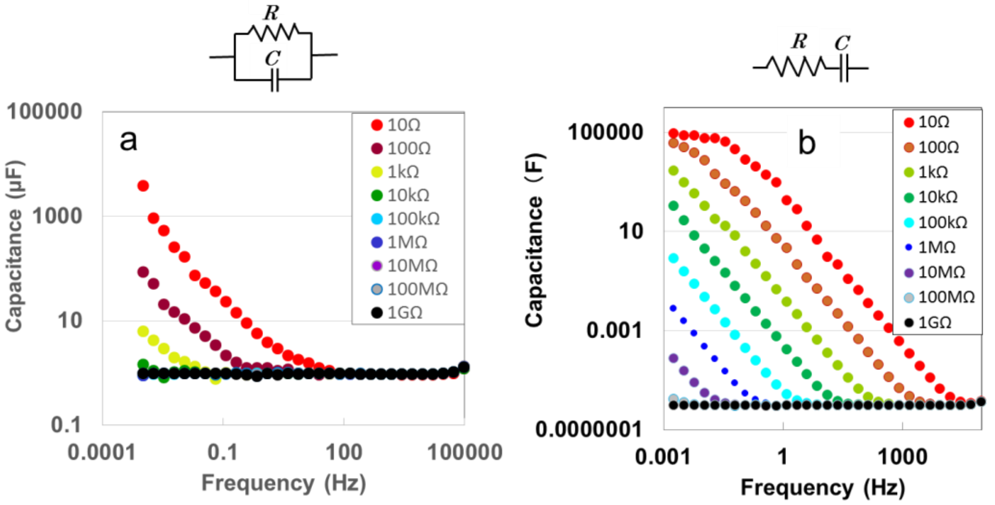

Fig. S2 Resistance and frequency dependencies of capacitance in parallel (**a**) and series circuits (**b**).

3. Comparison with surface structures of ATO and APP

We compared the surface structures of ATO and APP[6] using the noncontact atomic force microscope. Topography images of both surfaces are presented at Fig. S3, along with their physical properties. Both pictures are characterised by nanometre-sized uneven surface, the largest work functions for inorganic and organic substances, and higher resistivity.

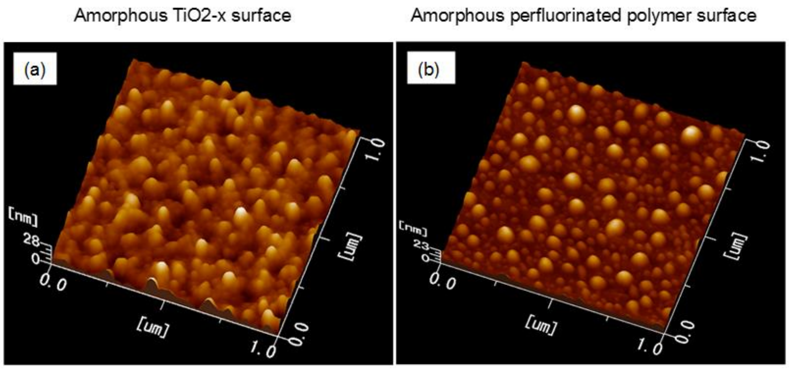

	Amorphous TiO2-x surface	Amorphous perfluorinated polymer surface
Specific weight	3.878	2.03
Glass transition point	555 K (Ti-Ni-Si)	4.71 K
Pore distance	35.4 nm	14.0 nm
Work function	5.53 eV	10.13 eV
Electric resistivity	10.5 TΩcm	48.1 TΩcm
Electron absorbed element	Ti	F
Quantum size effect	greater	greatest

Fig. S3 AFM images of ATO (**a**)and APP (**b**) surfaces.

4. Device fabrication by MEMS method

The device fabrication method by MEMS using ribbon specimens is presented by Fig. S4.

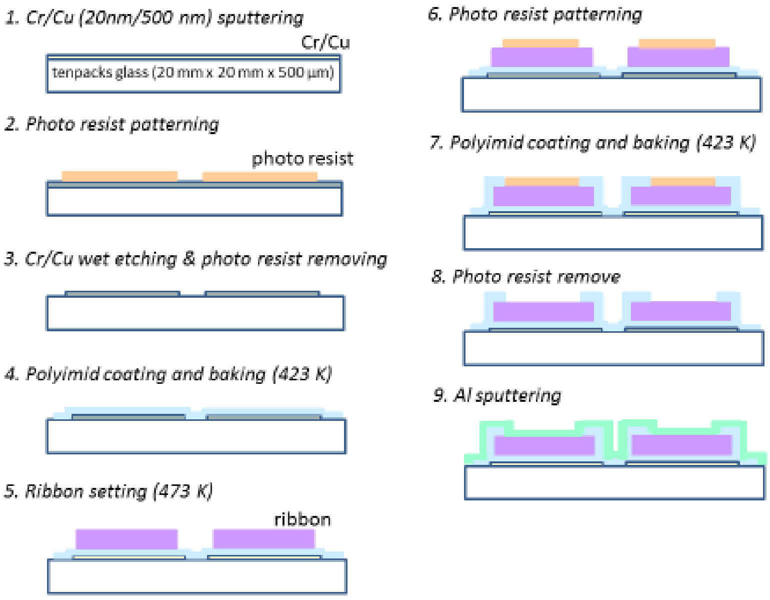

Fig. S4 Device fabrication process by MEMS method.

5. *Parallel connection of small capacitors*

In order to enhance charged amount for electrical storage, we connected fabricated devices (a schematic view is Fig. S5**a**) in parallel (Fig. S5**b**). Fig. S5**c** presents parallel connection of small capacitors.

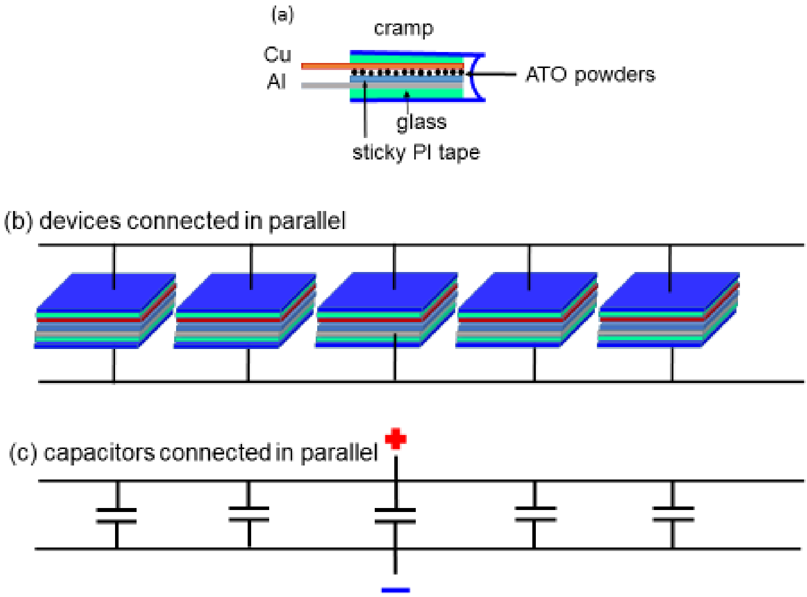

Fig. S5 (**a**) schematic view of a fabricated device, (**b**) schematic view of devices connected in parallel. (**c**) parallel circuit for capacitors.

6. Calculating the electrostatic potential of Ti on an uneven surface with nanometre-sized convexities using the Thomas-Fermi statistic method

Our interest lies in studying the electrostatic adsorption of titanium atoms centering the ATO with nanometre-sized convexities on an uneven surface with the quantum-size effect in view of the Thomas-Fermi (TF) electronic screening theory. The TF model has been applied to approximate calculations of potential fields and charge densities in elements as a function of lattice spacing. To the best of our knowledge, however, no detailed investigation has been conducted into the quantum-size effect for electric storage.

In general, nanoparticles with a particle size below 100 nm are characterised by a significant paucity in the ratio of chemical bonds in the particle surface. This suggests an increase in free electrons coming from the outer *s* and *p* subshells and resulting in a

relative decrease in the inner subshells in nanoparticles. This physical picture explains the lattice expansion of nanoscale compound particles from the neutralisation of the screening effect cause by the decreased binding-electron ratio [S2]. By reverse analogy, then, we can calculate the electrostatic potential and the electronic pressure of the titanium atoms centering the ATO.

When we assume that the convex portion is almost a half-sphere, the ratio η of the topmost atomic layer volume V to the half-sphere volume V increases as the half-sphere diminishes in size:

$$\eta = V'/V = 2\pi R^2 r/(4/6)\pi R^3 = 3r/R, \tag{1}$$

where r and R are radii of the layer atom and the sphere, respectively. Because the outermost bonding of the surface atoms is missing, the missing electrons are apparently free. The ratio of free electrons to binding electrons increases as the size decreases, suggesting a relative decrease in the binding electrons. Because the free electrons squeeze into the inner binding-electron region in nanometre-sized metallic particles [S3], the density ρ of the electrons associated with rigid bonding is calculated as follows:

$$\rho = \rho_0 (1+\eta), \tag{2}$$

where ρ_0 is the electron density of the "bulk" atom.

The following relation derives the screening length $1/\lambda$ [S4]:

$$\lambda^2 = 4\rho^{1/3}/a_o, \tag{3}$$

where $a_o = h^2/me^2$ is the radius of the first Bohr orbit of a hydrogen atom. For many-electron atom, the TF theory [S5] gives (in atomic units)

$$V(r) = -Ze^2\Phi(r/b)/r, \tag{4}$$

where $V(r)$ is the potential energy, and Z is the atomic number. According to Eq. (4), the screened Coulomb potential $\varphi(\mathrm{r})$ is written in the following form:

$$\varphi(\mathrm{r}) = qe^{-\lambda r}/r. \tag{5}$$

When the screened Coulomb potential, the screening length, the electron density, and the atomic radius of the "bulk" and nanosphere atoms is φ_o, $1/\lambda_o$, ρ_o, r_o, and φ_1, $1/\lambda_1$, ρ_1, r_1, respectively, we can solve

$$\varphi(r_o) = \varphi(r_1) \tag{6}$$

for r_1 under an electrostatic equilibrium in potential at r_o and r_1, using the TF table [S6] $\varphi_0(x)$.

We then consider an electronic contribution for the electric storage in terms of the bonding character of titanium ions, using their electrostatic potential. Because outer electrons in titanium ions are unsettled in the electronic structure with discrete permitted energies, several free electrons can move around the titanium ions. According to ideal gas model, the pressure of the material arises almost entirely from the electrons,

both because they outnumber the nuclei and because of their relatively small mass [S7]. In electronic kinetic theory, the relation between electronic pressure P and the total number of electrons per unit volume n is given as follows:

$$P = \frac{1}{5}\left(\frac{3}{8\pi}\right)^{\frac{2}{3}}\frac{h^2}{m}n^{\frac{5}{3}}. \qquad (7)$$

The number n is related to effective potential energy U of the outer electrons as follows:

$$n = \frac{8\pi}{3h^3}(-2mU)^{\frac{3}{2}}. \qquad (8)$$

Eqs. (7) and (8) can then be combined as follows:

$$P = \frac{8\pi}{15h^3}m^{\frac{3}{2}}(-2U)^{\frac{5}{2}}, \qquad (9)$$

where h is a Plank constant and m is the mass of the electron.

From Eqs. (6) and (9), we can calculate the electrostatic potential and the induced outer electronic pressure of the titanium atoms centering the ATO.

References

[S1] Fukuhara, A. and Sugawara, K., Anodic oxidization of Ti-Ni-Si amorphous alloy ribbons and their capacitive and resistive properties, *Thin Solid Films*, 595, 1-4 (2015), DOI:10.1016/j.tsf.2015.10.041.

[S2] Fukuhara, M. Lattice expansion of nanoscale compound particles, *Phys. Lett. A.* **313**, 427 (2003).

[S3] Paulus, P. M. *et al.* Surface and quantum-size effects in Pt and Au nanoparticles proved by ^{197}Au Mössbauer spectroscopy, *Phys. Rev. B.* **64**, 205418 (2001).

[S4] Kittel, C. *Introduction to Solid State Physics*, 4th edition (Wiley, 1971), p. 279.

[S5] Schwinger, J. Thomas-Fermi: The second correction, *Phys. Rev.*, **24**, 2353-2361 (1981).

[S6] Condon, E. U. & Odabaşi, H. *Atomic Structure* (Cambridge University Press, London, 1980), p. 454.

[S7] Hamann, S. H. *Physico-Chemical Effects of Pressure* (Butter Worths Scientific, London, 1957), p. 59.
